# Low concentrations of vorinostat decrease EB1 expression in GBM cells and affect microtubule dynamics, cell survival and migration

**DOI:** 10.18632/oncotarget.27892

**Published:** 2021-02-16

**Authors:** Thomas Perez, Raphaël Bergès, Hélène Maccario, Sarah Oddoux, Stéphane Honoré

**Affiliations:** ^1^Aix-Marseille University, CNRS, INP, Institute of NeuroPhysiopathology, Marseille, France; ^2^APHM, Hôpital de la Timone, Service Pharmacie, Marseille, France

**Keywords:** vorinostat, glioblastoma, EB1, microtubule, tubulin

## Abstract

Glioblastoma multiform (GBM) is the most frequent primitive brain tumor with a high recurrence and mortality. Histone deacetylase inhibitors (HDACi) have evoked great interest because they are able to change transcriptomic profiles to promote tumor cell death but also induce side effects due to the lack of selectivity. We show in this paper new anticancer properties and mechanisms of action of low concentrations of vorinostat on various GBM cells which acts by affecting microtubule cytoskeleton in a non-histone 3 (H3) manner. Indeed, vorinostat induces tubulin acetylation and detyrosination, affects EB stabilizing cap on microtubule plus ends and suppresses microtubule dynamic instability. We previously identified EB1 overexpression as a marker of bad prognostic in GBM. Interestingly, we show for the first time to our knowledge, a strong decrease of EB1 expression in GBM cells by a drug. Altogether, our results suggest that low dose vorinostat, which is more selective for HDAC6 inhibition, could therefore represent an interesting therapeutic option for GBM especially in patients with EB1 overexpressing tumor with lower expected side effects. A validation of our hypothesis is needed during future clinical trials with this drug in GBM.

## INTRODUCTION

Glioblastoma multiforme (GBM) is the most aggressive brain tumor [1] with a median survival of approximately 14 months with treatment [2]. Currently, the standard first-line treatment of GBM is based on surgical excision followed by the *Stupp* protocol [3]. However, some patients do not respond to treatment because of the GBM resistance to the ionizing rays of radiotherapy and to the action of chemotherapy. Concerning temozolomide, more than half of patients do not respond due to the overexpression of DNA repair enzymes, like the *O*^6^-methylguanine transferase [4–7], it is therefore urgent to find new therapeutic strategies. Histone deacetylase inhibitors (HDACi) are drugs that target the epigenetic of tumor cells [8]. HDACi have demonstrated anti-cancer properties *via* various mechanisms, such as cell-cycle arrest, inhibition of angiogenesis, activation of apoptosis pathway and cell death, production of reactive oxygen species [9]. Among them, vorinostat, also called SAHA (Suberanilo-hydroxamic acid), was approved by FDA in 2006 for human diseases like the treatment of cutaneous manifestations in patients with cutaneous T-cell lymphoma. It has showed anti-cancer activities like an up-regulation of the *p21* tumor suppressor gene, G_1_ cell-cycle phase arrest [10] and tumor cell autophagy induction [11]. Vorinostat is known as a non-selective HDACi and preclinical and clinical studies have shown beneficial effects in GBM [12]. Indeed, phase II studies in GBM has shown that this compound is well tolerated but has moderate antitumor activity [13, 14] and request further larger studies [12]. In 2018, a phase I/II study combined vorinostat and temozolomide in GBM patients. While the study was not conclusive for its primary efficacy end point, the authors found that vorinostat resistance and sensitivity signatures by RNA expression profiling of baseline tumors, had a positive correlation with overall survival and progression free survival for a subgroup of patients [15]. This strongly showed a real gain of vorinostat in some subpopulation. However, all of this works observed vorinostat effects using as rational end point the acetylation of histone 3 and 4 [10] the main target of class I HDAC 1, 2 and 3. However, this effect requires high doses of vorinostat and sometimes conduces to unanticipated toxicity in association with erlotinib (https://clinicaltrials.gov/ Identifier: NCT01110876). Vorinostat, while non-selective, preferentially inhibits HDAC 6 [16] which cellular target is acetylated tubulin. In this study, we were interested in effects of low doses of vorinostat on GBM cells microtubular system. Microtubules (MT) are formed by the assembly of α- and β-tubulin heterodimers. They contribute to cell morphology, motility, cellular transport processes, and cell division but also play a key role in neoangiogenesis and tumor progression [17]. The microtubular network constantly adapts to cellular needs and may be composed of very dynamic or more stable MT. To regulate their diverse functions in a spatio-temporal manner, MT are subjected to numerous reversible post-translational modifications [18]. MT are tubulin polymers that stochastically alternate between growth and shortening episodes, interrupted by periods of apparent stability. During cell migration, MT are mostly located and stabilized at the leading edge and displayed tubulin post-translational modifications such as tubulin detyrosination [19, 20]. For all these reasons MT are one of the most crucial targets for anti-cancer drugs. MT targeting agents (MTAs), which suppress MT dynamics [21, 22] are widely used for treatment of many human cancers.

Many studies have demonstrated the capital role of EB1 in cell migration [23–25]. EB1 belongs to the +TIPs (plus-end tracking proteins) family, that specifically bind MT (+) ends and control their dynamics [26–29]. EB1 is as a key player in the regulation of the MT dynamics, since it has been highlighted to proceed as a loading factor for other proteins that interact with MT, including those responsible for the MT stabilization at the cell cortex [30, 31]. Moreover, our team showed the impact of EB1 overexpression in GBM tumor progression *in cellulo* and its potential as a marker of response to MTAs [32, 33]. In GBM patients, overexpression of EB1 is a bad prognostic factor [32].

Here, we thus investigated the non-histone dependent effects of low doses of vorinostat on GBM cells behaviors and on microtubular system.

## RESULTS

### Vorinostat inhibits glioblastoma U87-MG, U87-P0 and U87-P11, GL261 and GBM6 cell survival

**Figure 1:**
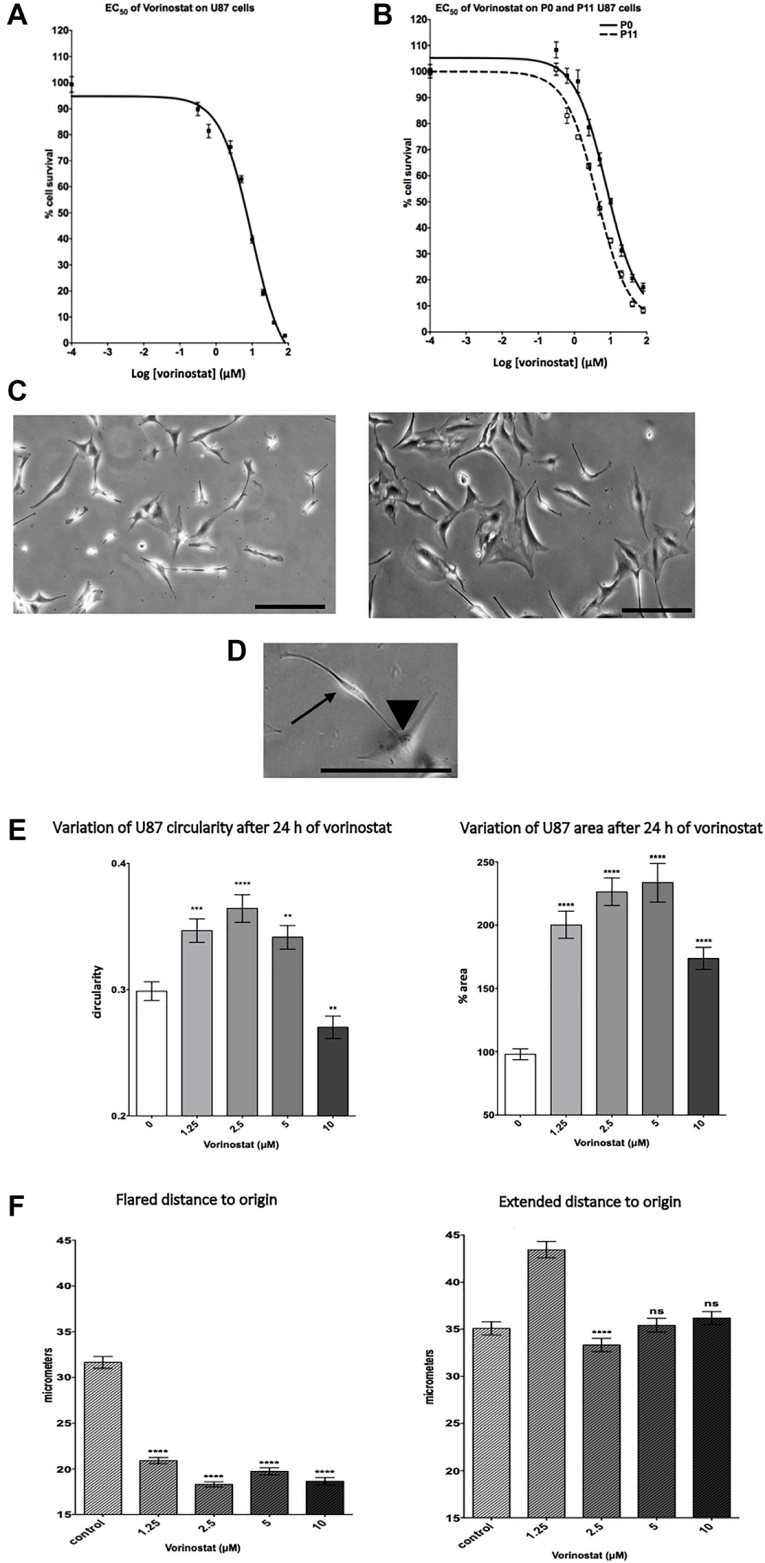
Vorinostat changes morphology and inhibits U87-MG glioblastoma cell migration and survival. (**A**) Dose response curves of the cytotoxicity of vorinostat in U87-MG cells. At least three independent experiments were performed. Results are expressed as mean ± SEM. (**B**) Dose response curves of the cytotoxicity of vorinostat in overexpressing-EB1 U87 P11 in comparison with control U87 P0 cells. At least three independent experiments were performed. Results are expressed as mean ± SEM. (**C**) Representative images of U87-MG cells after 24 hours treatment with vorinostat 5 μM, bar = 0,1 mm. (**D**) Representative focus image on U87-MG extended cell (arrow) and flared cell (arrow head) in cellular culture with vorinostat 5 μM, bar = 0,1 mm. (**E**) U87-MG cell lines circularity and area at each vorinostat doses considered. Histograms show circularity (left side) and area variation (right side). Results are expressed as mean value ± SEM, compared to control by unpaired two-tailed *t*-test. (**F**) Cell migration measured by 2D video microscopy; extension distance and flared distance to origin (micrometers). Histograms show migration variation mean value ± SEM, U87-MG cell lines at each vorinostat doses considered, compared to control by unpaired two-tailed *t*-test. (^*^) indicates significant differences from control: ^*^ *p* < 0.05; ^**^ *p* < 0.005; ^***^ *p* < 0.001, ^****^ *p* < 0.0001 n.s.: non-significant.

Dose-response cytotoxicity assays of vorinostat were conducted on human GBM cell line (U87-MG), murine GBM cell line (GL261) (Figure 1A, Supplementary Figure 1A). The drug concentrations necessary to reduce viability by 50% (EC_50_) were determined after 72 h treatment. Vorinostat appeared to be cytotoxic at micromolar concentration. We obtain an EC_50_ of 9.7 ± 0.10 μM on U87-MG and 6.3 ± 1.45 μM on GL261. Moreover, we also tested the effect of vorinostat on glioblastoma cancer stem-like cells (GBM6) and found an EC_50_ of 0.43 μM ± 0.11. In parallel, vorinostat effect on overexpressing-EB1 U87-MG cells (P11) survival was tested for comparison with their own control (P0) (Figure 1B). Interestingly, EC_50_ of vorinostat was decreased by 46% in EB1 overexpressing cells compared with control cells (4.16 ± 1.08 μM vs 7.70 ± 1.13 μM respectively).

### Vorinostat affects U87-MG cells morphology

Figure 1C shows cellular morphology of U87-MG glioblastoma cancer cells cultured with or without 5 μM vorinostat for 24 h. We observed that U87-MG cells seems, in majority, to be more flared under vorinostat treatment in comparison to the control which appeared more extended (Figure 1C and 1D). The mean corresponding cellular spreading parameters (circularity and cytoplasm area variation) are shown in Figure 1E. After 24 hours treatment, low doses vorinostat (1.25; 2.5 and 5 μM) increases circularity (+15%, +22%, and +19% respectively) and cell areas (+102%, +128%, and +135% respectively). Surprisingly, the effects appeared less important at more important dose (10 μM).

### Vorinostat inhibits U87-MG cell migration

The change in morphology of the cell induced by vorinostat impacted the cell migration process mesured by 2D videomicroscopy (Figure 1F). Indeed, vorinostat decreases cell migration measured by the distance to origin of U87-MG flared cells (–58% with 2.5 μM vorinostat) whereas there is no effect on remaining extended cells. Effect of vorinostat was also assessed by using a transwell assay on GL261 and GBM6. Vorinostat significantly decreased cell migration for GL261 (−22.4 ± 18% and −23.2 ± 16.1% at 2 μM and 4 μM) (Supplementary Figure 1B) and for GBM6 (−28% and −67% at 0.2 μM and 1 μM) (Supplementary Figure 3A). Our results clearly show that vorinostat decrease GBM cell migration.

### Vorinostat alters microtubules post-translational modifications and decreases EB1 expression

**Figure 2:**
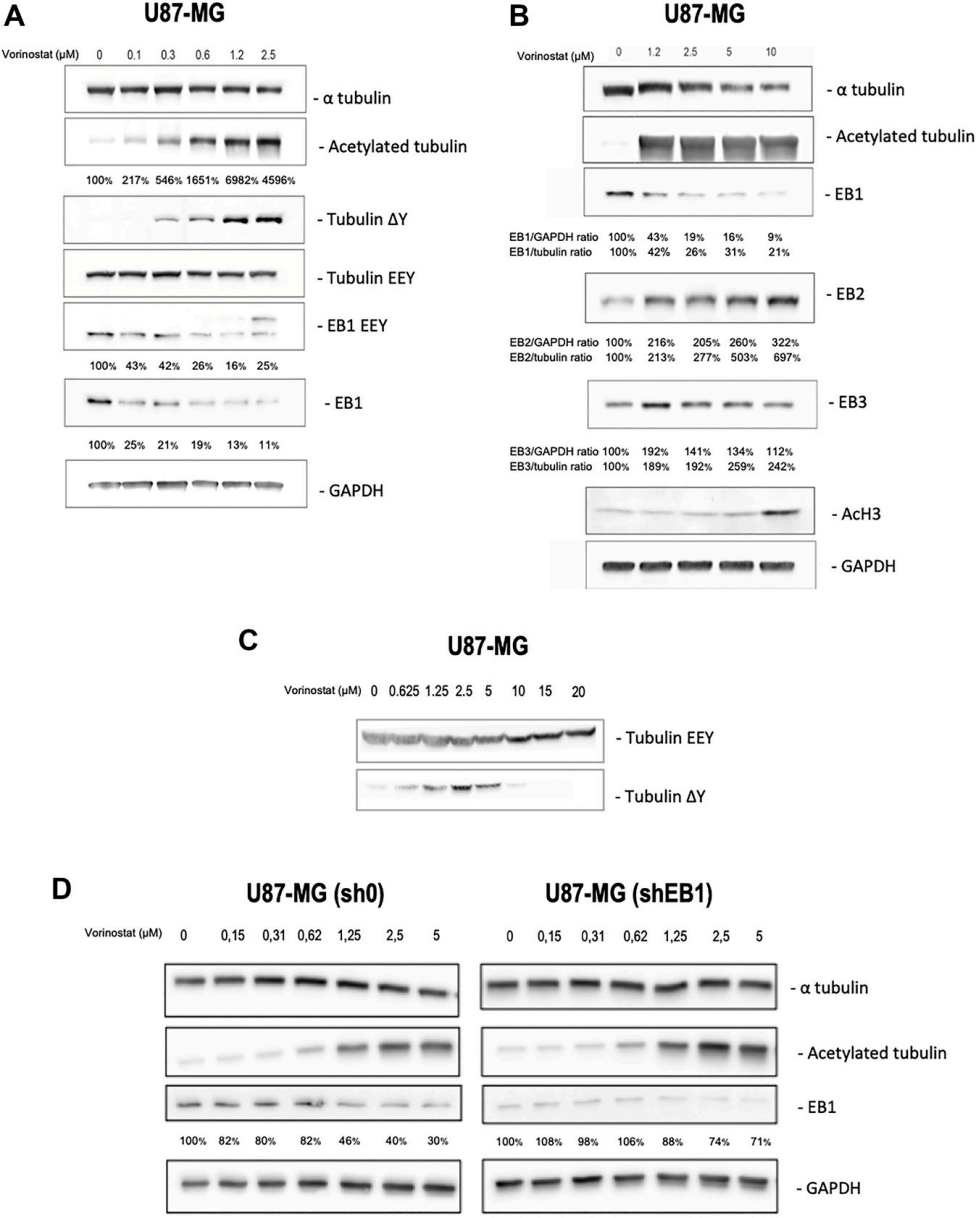
Vorinostat acts on microtubular system in an independent histone manner in U87-MG, U87-MG sh0 and U87-MG shEB1 glioblastoma cells. Analysis of tubulin, EB proteins, and PTM (post translational modification) level expression by Western blot on U87-MG cells treated for 24 h with vorinostat at various concentration in parallel with Histone H3 acetylation. (**A**) α tubulin, acetylated tubulin, tubulin ΔY, tubulin EEY, EB1 EEY and EB1 proteins level expression, 0 to 2.5 μM vorinostat. Ratios (%) acetylated tubulin/GAPDH, EB1 EEY/GAPDH and EB1/GAPDH, from at least three independent experiments are presented under the blots. (**B**) α tubulin, acetylated tubulin, EB1, EB2, EB3 and histone H3 proteins level expression, 0 to 10 μM vorinostat. Ratios (%) EB1/GAPDH, EB2/GAPDH and EB3/GAPDH and ratios (%) EB1/tubulin, EB2/tubulin and EB3/tubulin, from at least three independent experiments are presented under the blots. (**C**) Tubulin EEY and tubulin ΔY proteins level expression, 0 to 20 μM vorinostat. (**D**) α tubulin, acetylated tubulin and EB1 proteins level expression, 0 to 5 μM vorinostat, U87-MG sh0 et U87-MG shEB1. Ratios (%) EB1/GAPDH, from at least three independent experiments are presented under the blots.

In order to study the impact of vorinostat on tubulin and EB1, we carried out western blotting on U87-MG and EB1-underexpressing U87-MG cell clone treated with vorinostat or untreated (Figure 2). Vorinostat is known to inhibit HDAC6 leading to tubulin acetylation. Indeed, we found from that vorinostat increases acetylation of tubulin in a concentration-dependent manner from 0.1 μM to 10 μM. However, acetylation of histone 3 (AcH3) only occurred at 10 μM which suggest a non-histone regulation at concentration below this concentration, due to the preferential targeting of vorinostat on HDAC6. Concomitantly, for low doses of vorinostat (0.3 to 5 μM) we observed an increase of tubulin detyrosination which also appeared to be concentration dependent in this range. These effect on both tubulin acetylation and detyrosination were confirmed by immunofluorescence staining (Supplementary Figure 2). However, we observed a opposite effect on tubulin detyrosination at concentration ≥ 10 μM (Figure 2C), suggesting an effect of vorinostat on others cellulars targets than HDAC6 in accordance with the effect of histone 3 acetylation. Finally, vorinostat appeared to decrease α-tubulin expression for concentration ≥ 5 μM.

Interestingly, we found for the first time that vorinostat affects the expression of End Binding proteins (EBs). Indeed, EB1 expression level, measured using both an EB1 antibody (KT51) or using the YL½ antibody that recognize tyrosinated at 30 kDa), was strongly decreased in U87-MG cells starting at the lowest dose (0.1 μM) (Figure 2A and 2B). Such new effect was confirmed on both murine GL261 (Supplementary Figure 1C) and in cancer stem like cells GBM6 (Supplementary Figure 3B). In parallel of this reduction, EB2 and EB3 seem to compensate for EB1 (Figure 2B). Finally, reduction of EB1 expression was more pronounced in cells with higher level of EB1 (Figure 2D).

### Vorinostat decreases EB3 comets and alters microtubule dynamics

**Figure 3:**
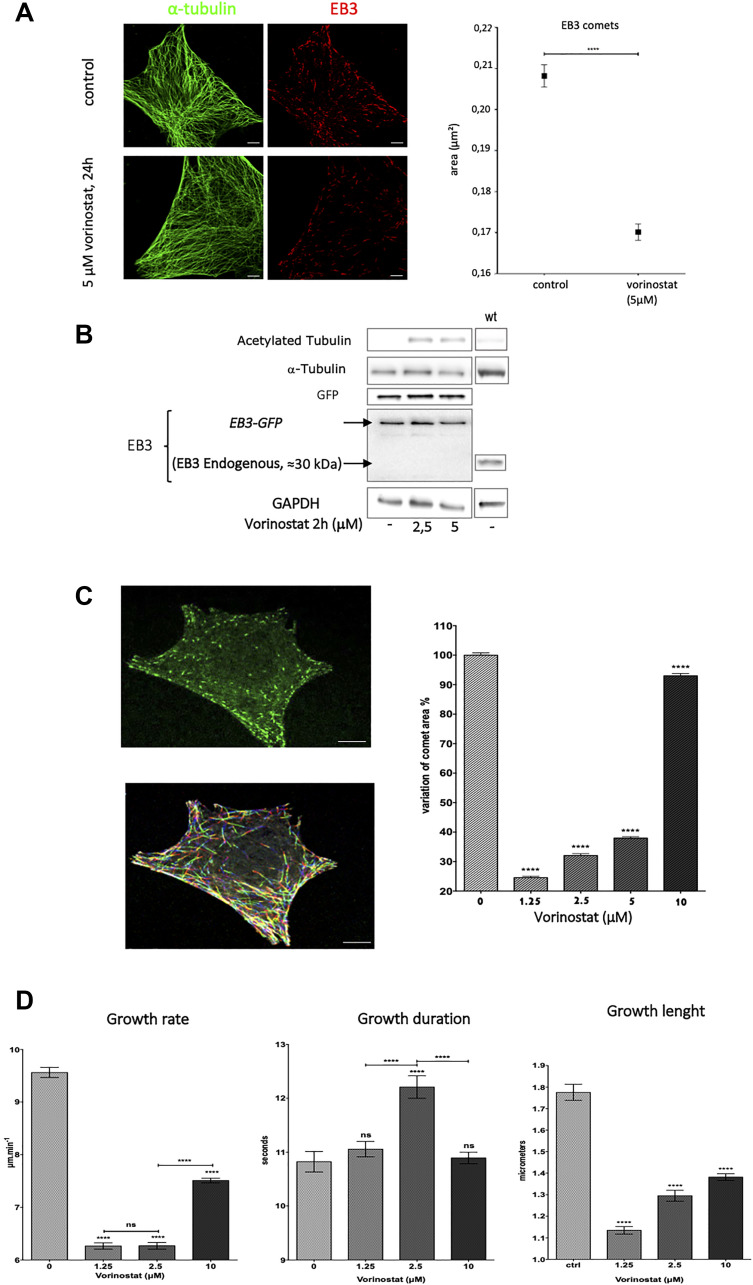
Vorinostat deacreases EB3 comets area and suppress microtubule dynamics in U87-MG glioblastoma cells. (**A**) Immunofluorescence staining of α-tubulin (green) and endogenous EB3 (red) in U87-MG cells with 24 hours 5 μM vorinostat and control (left panel) and vorinostat effect on EB3 comet longer and area, ^****^ *p* < 0.0001, vs control, Student’s *t*-test, (right panel), bar = 5 μm. (**B**) Analysis of EB3 protein level expression by Western blot of U87-MG cells transfected by EB3-GFP treated after 24 h of treatment with vorinostat 2 μM. (**C**) Representative image from time-lapse videomicroscopy of U87-MG cells transfected with EB3-GFP cells (top), representative image of EB3 comet trajectories in cells obtained from ICY^®^ software (gradient of colors according time, bottom) and variation of comet area under vorinostat, bar = 10 μm (right panel). (**D**) Parameters of EB3-GFP dynamics. All values are expressed as mean ± SEM of comets tracks analyzed (n.s.: non-significant., ^****^ *p* ≤ 0.0001 each condition *vs* control, Student’s *t*-test).

Previous experiments showed that vorinostat altered End Binding proteins expression (EB1, EB2 and EB3) in GBM cells. We were interested on the impact on microtubule dynamics. We first determined the intracellular localization of EB3, a +TIP protein that controls microtubule dynamic instability and which expression level is not altered by vorinostat. Immunofluorescence microscopy unveiled a typical shape of EB3 with comet-like structures at the (+) ends of MTs in U87 cell line (Figure 3A, left panel). Interestingly, we observed a decrease of endogenous EB3 comets area with 5 μM vorinostat (Figure 3A) suggesting an alteration of microtubule plus end dynamics.

We thus transfected U87-MG cells with EB3-GFP in order to track microtubule plus end dynamic behavior. We first verified the expression level of both endogenous EB3 and EB3-GFP in transfected cells by Western blot (Figure 3B). Interestingly, after transfection with EB3-GFP plasmid, we did not detect endogenous EB3. The analysis of EB3-GFP comet area in transfected cells confirmed the result observed with endogenous EB3. Indeed, we found a decrease of EB3 comet area for low concentration of vorinostat ≤ 5 μM. Consistently with our previous observation, at 10 μM vorinostat, the effect on EB3 comets was no more observed (Figure 3C right panel).

We performed live imaging confocal microscopy to follow all MT (+) ends positions in the cytoplasm of U87-MG cells transfected with EB3-GFP treated by vorinostat. Using plusTipTracker software, we tracked EB3 comets dynamics and analyzed dynamic instability parameters. Treatment with vorinostat significantly decreased the mean microtubule growth rate by –35.6% from 9.6 μm/min in control cells to 6.2 μm/min for 1.25 and 2.5 μM vorinostat. As expected, the effect was less important at 10 μM (*p* < 0.001). Analysis of microtubule growth length and duration showed that vorinostat mainly decreased growth excursion length (Figure 3D, right panel). These results indicate that vorinostat, like microtubules targeting agent, are able to alter EB3 accumulation at microtubule plus end and alter microtubule dynamics in GBM cells.

### Vorinostat induced decrease in EB1 expression is restricted to endogenous protein and independent of proteasome

**Figure 4:**
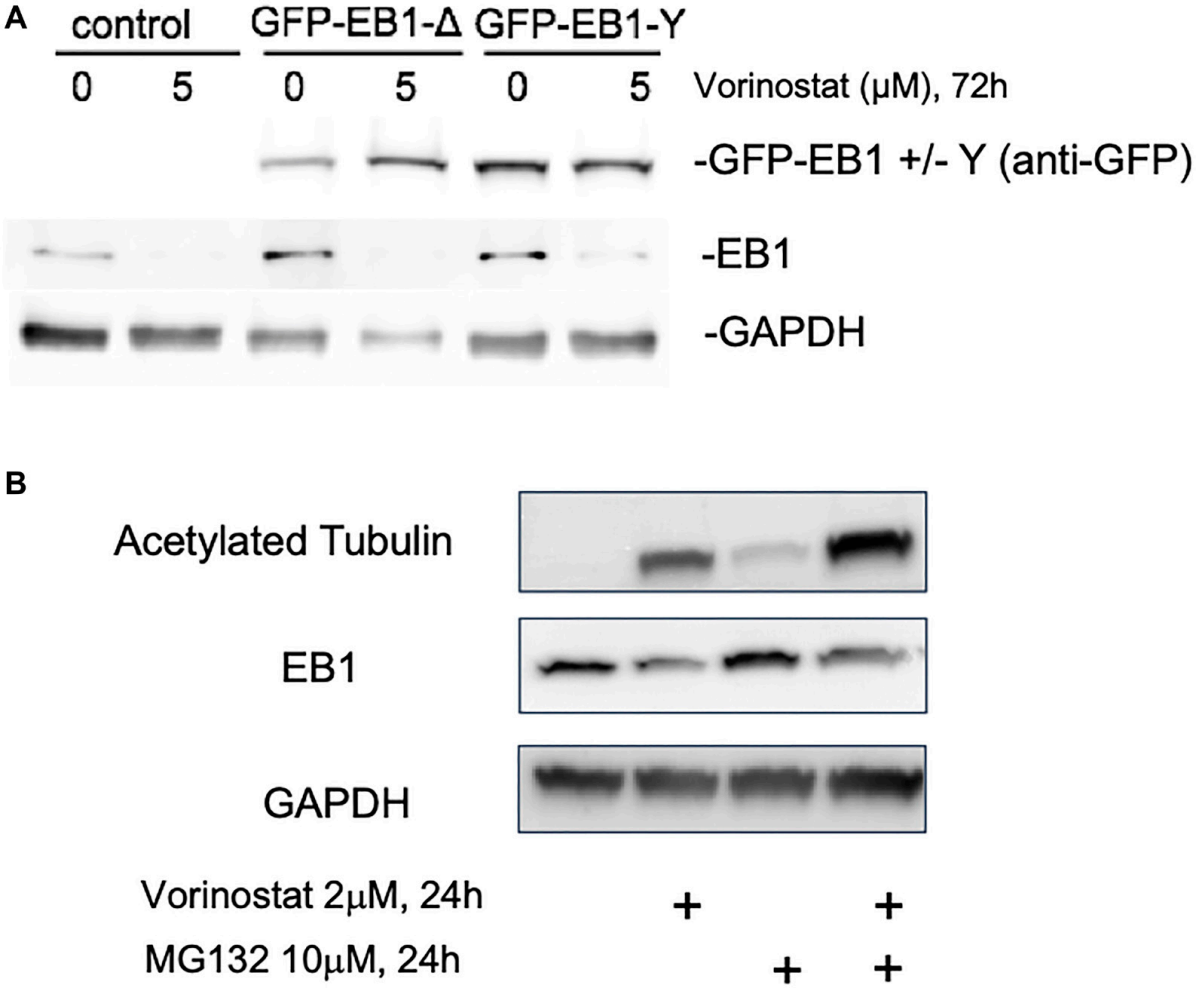
Vorinostat changes U87-MG endogenous EB1 expression but not GFP-EB1. (**A**) Analysis of tyrosinated tubulin, EB1, GFP-EB1 tyrosinated and detyrosinated level expression by Western blot of U87-MG cells transfected by GFP-EB1-detyr and GFP-EB1-tyr, treated after 24 h of treatment with vorinostat 5 μM. (**B**) Analysis of acetylated tubulin and EB1 protein level expression by Western blot of U87-MG cells, treated after 24 h of treatment with vorinostat 2 μM and/or MG132; 2 μM.

To investigate whether the apparent decrease in EB1 expression level by vorinostat is due to an increased degradation or to a decreased expression, we first tested by western-blotting in U87-MG cells compared to endogenous EB1, the effect on GFP-EB1 exogenous construct with non-endogenous promotor regulation for expression (GFP-EB1-tyr) (Figure 4A). We also used the GFP-EB1-detyr construct in order to detect any effect with the tyrosination/detyrosination cycle of EB1 [19]. We show that there is no reduction for both constructs while in the same time we find a depletion for endogenous EB1 when treated by vorinostat 5 μM for 72 h, suggesting an effect on EB1 expression but not on degradation (Figure 4B). Moreover, we found that MG132, a proteasome inhibitor, did not prevent vorinostat-induced decrease of EB1, suggesting a proteasome independent process. Together, our results more likely suggest a direct effect on EB1 expression which remain to be explored.

## DISCUSSION

Several properties of GBM, like proliferation, migration, angiogenesis, invasion, and resistance to apoptosis, are targeted by HDACi by an epigenetic and non-epigenetic regulation [34]. HDACi can modify transcriptomic profiles to instigate cancer cell death and this activity occurs at micromolar concentrations, already shown for this class of drug in other cancer cells like renal cancer cells [35], lung cancer cells [36] or colon cancer cells [37]. To date, clinical trials testing the safety and efficacy of vorinostat in GBM patients, used this epigenetic mechanism with acetylated H3 and H4 as endpoint markers [14].

In this study, we show that vorinostat altered GBM cell morphology and decreases human, murine and cancer stem-like cells GBM survival and migration. Interestingly, such anticancer effects mainly occurred at low concentrations of vorinostat that did not increase acetylated histone H3 (AcH3), which is known to be mediated by class I HDAC 1 and 3, the latter being overexpressed in GBM and associated with poor survival [32]. At such low concentrations, vorinostat more likely inhibit HDAC6, its preferential target [16]. Our results are in agreement with those of *Wang and al* showing that inhibition of HDAC6, which is overexpressed in GBM, by specific inhibitors display substantial anti-GBM activity [38].

HDAC6 a class IIb HDAC, deacetylates several substrates, including α-tubulin whether in the nucleus and in the cytoplasm. Indeed, by shuttling between these two locations, HDAC6 regulates epigenetic and non-epigenetic mechanisms, reviewed in [39].

Inhibiting HDAC6, vorinostat increased tubulin acetylation as it has already been shown [40] but have also other impacts on the microtubule cytoskeleton. Interestingly, we show that low concentrations of vorinostat (< 10 μM), also induced tubulin detyrosination, concomitantly with tubulin acetylation. The tubulin detyrosination is regulated by the tubulin carboxypeptidases, recently identified as vasohibins (VASH1 and 2) [41]. Both tubulin acetylation and tubulin detyrosination are correlated with less dynamic MT (longer-lived MT), while more dynamic microtubules are found to be mainly non-acetylated and tyrosinated [42–44]. Indeed, tubulin detyrosination is now well known to promotes microtubule stability [44–47]. In our context, tubulin detyrosination in the presence of the HADC6 inhibitor, vorinostat, may thus be more likely associated with a decrease in dynamic microtubules independently of tubulin acetylation. Altogether our results demonstrated that vorinostat, beside its effects on the “histone code”, also modify the “tubulin code” [48].

Moreover, it was shown that both tubulin detyrosination and EB1 detyrosination, are able to impair accumulation of CAP-Gly proteins at growing MT (+) end, thereby decreasing MT dynamics and associated cell functions [49, 50]. Interestingly, our results demonstrate that vorinostat modify the microtubule plus end stabilizing cap (evidenced using EB3 staining) and suppresses microtubule dynamics instability. This effect on microtubule dynamic instability is almost similar to mechanism of action of microtubule targeting agents, known for their anti-cancer properties [17, 51]. Such effect on MT dynamics by HDAC6 inhibition may involves an interaction between HDAC6 and EB1 [52].

However, we show for the first time a dramatic concentration dependent reduction of EB1 expression by low concentration vorinostat in all studied GBM cell lines, including glioma stem-like cells (GBM6). This strongly contrast with microtubule targeted agents, since they alter both EB comets and microtubule dynamics but without affecting EB1 expression.

This result is of great importance because of several reasons: i) such effect was never evidenced for any drug to our knowledge, ii) the depletion occurred at low dose of vorinostat which did not induce AcH3, which suggest a non-histone 3 regulation, iii) the effect appeared to be very sensitive because it’s occur since the lowest dose, concomitantly with tubulin acetylation for U87-MG / U87 Sh0/ShEB1 and GBM6 cells and before tubulin acetylation in murine GL261 cells, iv) the effect appeared linked to EB1 expression level, v) the effect is proteasome-independent and only affect endogenous EB1 suggesting a direct transcriptional effect on EB1 expression via HDAC6 epigenetic regulation [39]. Interestingly, we found a mirror regulation of EB2 expression but not EB3 suggesting that EB1 and EB2 are regulated in an opposite way by vorinostat.

Surprisingly, in contrast to tubulin acetylation and decreased EB1 expression, we observe a switch on the effect tubulin detyrosination at concentration of vorinostat ≥ 10 μM when Histone 3 became acetylated. Indeed, detyrosinated tubulin completely disappeared and level of tyrosinated tubulin strongly increased. Moreover, effects on the microtubule stabilizing cap and microtubule dynamics were strongly attenuated. These results highlight a different microtubule regulation by high concentrations of vorinostat, probably through transcriptional effects linked to class I HDACs.

Finally, we previously showed that EB1 favors GBM cell migration and proliferation in U87-MG cells [19] which is confirmed in this study for GBM6 stem like cells. Some publications show the probable role of EB1 in tumorigenesis of several human cancers suggesting an oncogenic role [53–58]. Beyond its oncogenic role, we recently demonstrated the poor prognostic value of EB1 overexpression in GBM patients [32]. Interestingly, we found that vorinostat cytotoxicity was EB1 expression level dependent on the U87-MG P0 and P11 cells. The discovery of a potential drug that dramatically reduces the expression of such oncogene and bad prognostic marker in GBM patients is of great interest for clinical research and particularly for a subgroup of GBM patients with EB1 overexpressing tumors. In conclusion, our results open new research opportunities on vorinostat /HDAC 6 inhibitors in GBM which has currently limited therapeutic options.

## MATERIALS AND METHODS

### Chemical compounds

Vorinostat (Sigma-Aldrich) was solubilized in dimethyl sulfoxide (DMSO).

### Cell culture

U87-MG glioblastoma cell (human glioblastoma cells) was purchased from the American Type Culture Collection. Cells were cultured in EMEM with glucose and L-glutamine (Lonza), with 10% fetal bovine serum (Lonza), 1% (100U/mL) penicillin-streptomycin (Sigma-Aldrich). GBM6 (stem-like cells) were extracted in our team from GBM tumors patients and cultured as previously published [59]. GL261 glioblastoma cell (murine glioblastoma cells, National Cancer Institute, Charles River Labs) cultured in RPMI1640 + GlutaMAX (Gibco) with 10% fetal bovine serum (Lonza). All cells were maintained at 37 °C and 5% CO_2_ in a humidified incubator.

### Cell transfection

ShRNA plasmid that specifically knocked out human EB1 and negative shRNA control plasmid were purchased from Sigma-Aldrich. EB1 expression plasmid and negative control plasmid were purchased from Addgene. We previously generated U87 stable clones overexpressing EB1 (U87 P11) or underexpressing EB1 (U87 shEB1) and their relative control clones U87 P0 and sh0 with respective empty control vectors [32]. In U87 shEB, expression level was approximately 2 fold lower than in U87 control or U87-MG wild type cells. Moreover, in overexpressing EB1 clones U87 P11, EB1 levels were 7.4 fold higher than in U87 control or in U87-MG wild type cells.

### Analysis of cell survival

Cytotoxic effect of vorinostat on GBM cell lines was measured by using a colorimetric assay: MTT assay, based on the metabolic activity of mitochondria, which reflects cell viability. Twenty-four hours after seeding, cells were treated with several concentrations of vorinostat for 72 h. After treatment, 10 μL of MTT solution were added to each well and were incubated for 4 hours at 37°C. Formazan crystals were dissolved in 100 μL of DMSO and the absorbance was determined spectrophotometrically at 562 nm using an Elx800 universal microplate reader and data were analyzed with the Gen5 software. At least three independent experiments were conducted.

### Analysis of cell circularity and area

Images of cells (30 cells per condition) were collected with a microscope Nikon TE 2000 connected to a digital camera (Princeton Instruments). Circularity and area were extracted and quantified by image analysis using open-source tools, Image J software. Data are representative of three independent experiments.

### Random motility assay

Cells were seeded in culture plates, coated with fibronectin 10 μg/ml. One hour later, cells were either treated with vorinostat at several concentrations or untreated, and subjected to time-lapse video-microscopy. Images were recovered every 10 minutes during 10 h with a microscope Nikon TE 2000 connected to a digital camera (Princeton Instruments). Random motility data were determined as previously described [60]. Thirty cells per condition were tracked, for each experiment. At least, three independent experiments were conducted.

### Migration assay

Fifty thousand cells per well were added to the upper chamber of transwell migration chamber (Becton Dickinson). After 5 h of incubation, six randomly selected images were captured per condition and transmigrated cells were counted. Results were expressed as percentage of transmigrated cells compared with no treatment condition. For each condition, three independent experiments were performed.

### Immunofluorescence staining

Indirect immunofluorescence was performed with the anti-EB3 antibody (EPR11421(B), Abcam), anti-acetylated tubulin antibody (Merck millipore), anti-detyrosinated tubulin antibody (Abcam) and anti-mouse antibody Alexa 568 nm (Molecular Probes); and FITC-coupled anti-α-tubulin antibody (clone DM1A; Sigma-Aldrich). Images of cells were captured with Leica DM-IRBE microscope. All images were acquired using Metamorph software (Molecular Devices) at identical acquisition settings, and were processed using Image J software. Mean EB3 comet area was analyzed with Metamorph software on at least 10 representative cells per condition and 3 independent experiments were conducted. Data were expressed as mean ± SEM and statistical analysis was performed using Student’s *t*-test.

### Western blot analysis

Proteins were extracted from cell cultures and lysed with Laemmli sample buffer containing 2% SDS, 52.5 mM Tris-HCl and protease inhibitors (Roche diagnostics). Equal amounts of protein from each treatment were loaded into 12% SDS-polyacrylamide gels. Anti- EB1 (clone KT51, Abcam), anti-EB2 (Abcam), anti-EB3 (EPR11421(B), Abcam), anti-α-tubulin (Sigma-Aldrich), anti-acetylated tubulin (6-11B-1, Merck millipore), anti-detyrosinated tubulin (Abcam), and anti-GFP (Abcam), anti-GAPDH (Clone, source), anti-acetylated histone H3 (Merck millipore) and anti-mouse IgG-horseradish peroxidase (Jackson Immunoresearch) were used. YL½ antibody (Merck Millipore) was used to detect both tyrosinated tubulin (~ 50 kDa) and tyrosinated EB1 (~ 30 kDa). U87 cells were transfected with GFP-EB1 and Detyrosinated-GFP-EB1 plasmids [19] using lipofectamineTM 2000 system (Invitrogen) and left to incubator for 24–48 h. Visualization of protein bands was performed with a chemiluminescence detection kit (Millipore) and the chemiluminescent signal was acquired with a G:BOX imaging system (Syngene). Quantification of western blot bands was performed with Image J software.

### Analysis of microtubule dynamics

Microtubule dynamics were analyzed from EB3-GFP data as previously described [32]. Briefly, sixty thousand U87-MG cells per well were grown during 24 h on 8-well Labtek (Thermo Scientific) precoated 1 hour with fibronectin. Cells were then transfected with plasmid encoding EB3-GFP using lipofectamineTM 2000 system (Invitrogen) and left to incubator for 24–48 h. Before time-lapse microscopy analysis, U87-MG-EB3 cells were incubated with several concentrations of vorinostat for 4 hours. Time-lapse microscopy and image for MT dynamics experiments were performed with a Leica DM-IRBE. Sixty images per cell were acquired at 2 seconds intervals with a digital camera (Princeton Instruments). Analysis of MT dynamic instability was done by tracking plus-end EB3-GFP comets and were detected using the plusTipTracker software. Comet detection requires no user intervention, as the detection algorithm automatically estimates locally optimal thresholds. Tracking and inference of complete MT trajectories by plusTipTracker requires user-defined settings of several control parameters previously described [32]. To assess the consistency, we chose 3 metrics for comparative analyzes: mean growth rate, mean growth duration, mean growth length. A Student’s *t*-test was performed for each of these metrics. For each experimental condition, 5 to 8 cells were analyzed (400-1200 comet tracks per cell).

### Statistical analysis

Data are reported as mean ± standard error of the mean (SEM). All data were verified in at least 3 independent experiments. Cellular viability data were analyzed by Student’s *t*-test. Reported *p* values are two-sided, and only values of *p* < 0.05 were considered as significant. Asterisks indicate significant level versus control: ^*^ *p* < 0.05; ^**^ *p* < 0.005; ^***^ *p* < 0.001; ^****^ *p* < 0.0001. Statistical analyses were performed using Graph Pad-Prism 5 software (Graph Pad Software).

## SUPPLEMENTARY MATERIALS



## Abbreviations

GBM: Glioblastoma multiforme
HDACi: Histone deacetylase inhibitors
SAHA: Suberanilo-hydroxamic acid
MT: Microtubules
MTAs: MT targeting agents
EB1: End Biding protein 1
+TIPs: plus-end tracking proteins
EC50: effective concentration
AcH3: Acetylated Histone 3
DMSO: dimethyl sulfoxide
