# Supplementary material for: Low concentrations of vorinostat decrease EB1 expression in GBM cells and affect microtubule dynamics, cell survival and migration
Source: Oncotarget. 2021 Feb 16;12(4):304–15. doi: 10.18632/oncotarget.27892 (PMC7899546; doi:10.18632/oncotarget.27892)
Supplement: Supplementary file 1 [file oncotarget-12-304-s001.pdf]

Low concentrations of vorinostat decrease EB1 expression in GBM cells and affect microtubule dynamics, cell survival and migration

SUPPLEMENTARY MATERIALS

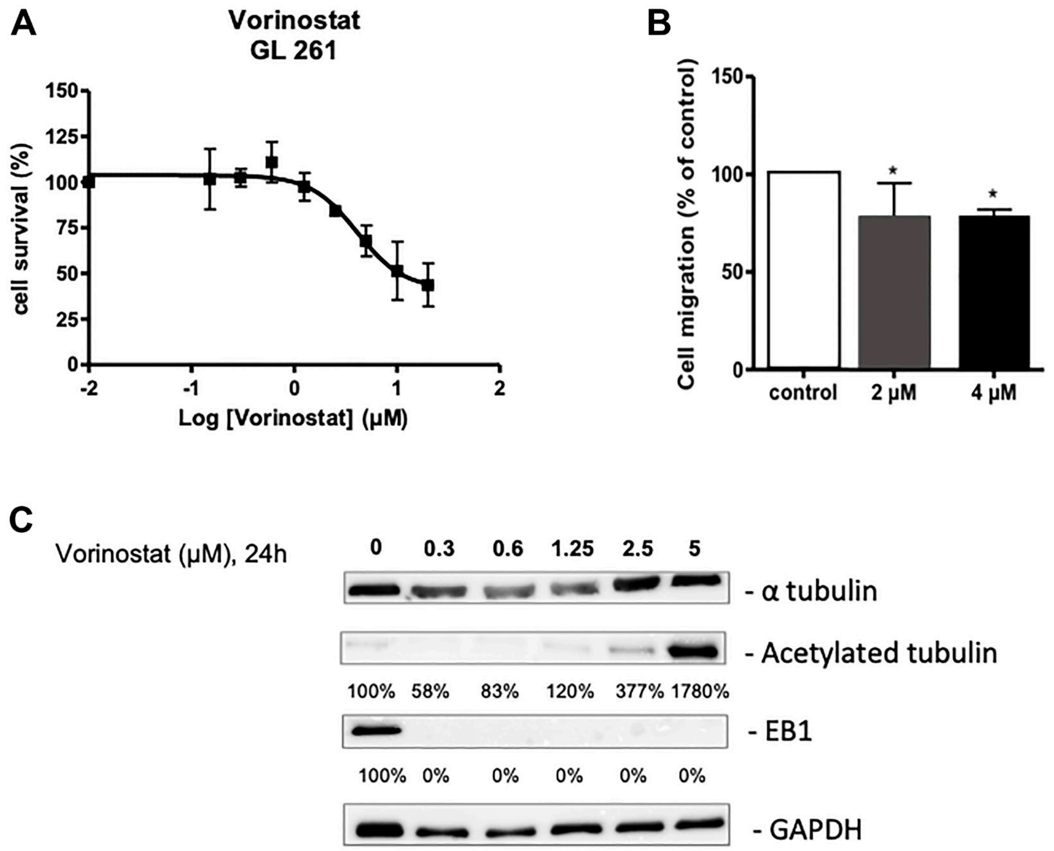

Supplementary Figure 1: Vorinostat inhibits murine glioblastoma cell migration and survival. (**A**) Dose response curves of the cytotoxicity of vorinostat on murine GL261 glioblastoma cells. At least three independent experiments were performed. Results are expressed as mean ± SEM. (**B**) Quantification of migration of GL261 cells by transwell migration assay, Bar ± SEM. (*) indicates significant differences from control: *$p < 0.05$. (**C**) Analysis of α tubulin, acetylated tubulin and EB1 proteins level expression by Western blot, of GL261 cells after 24 hours of treatment with vorinostat. Ratios (%) Acetylated tubulin/GAPDH and EB1/GAPDH, from at least three independent experiments are presented under the blots.

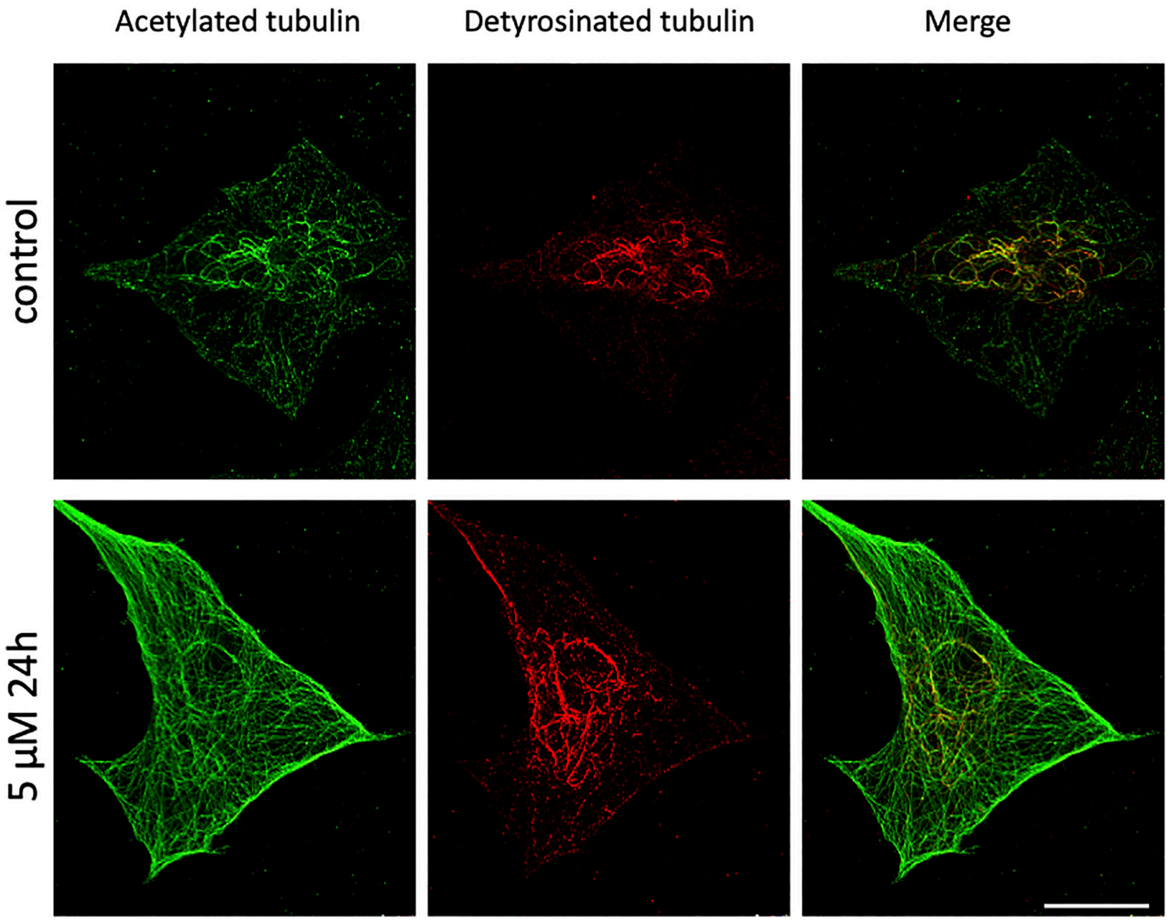

Supplementary Figure 2: Vorinostat induces tubulin acetylation and detyrosination. Immunofluorescence staining of acetylated tubulin (green) and detyrosinated tubulin (red) in U87-MG cells with 24 hours 5 μM vorinostat and control, bar = 10 μm.

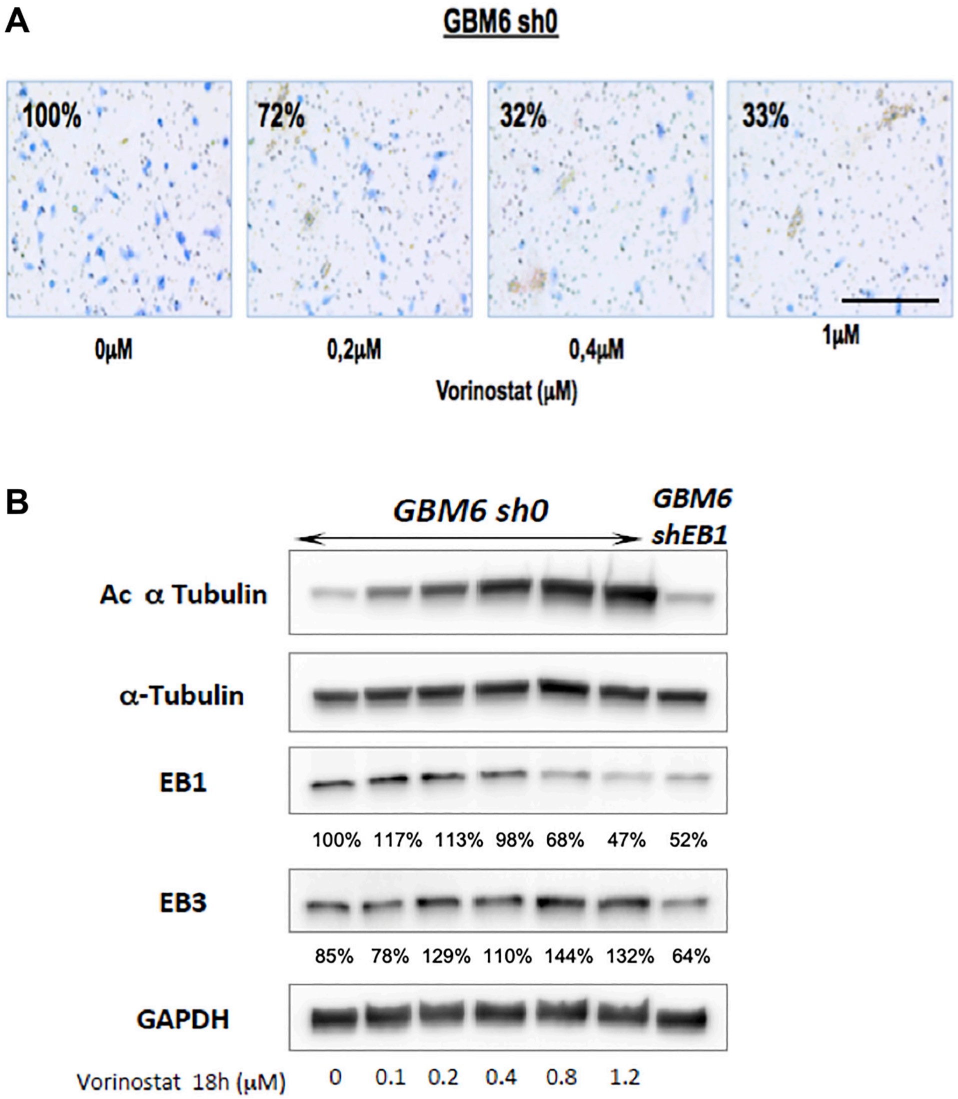

Supplementary Figure 3: Vorinostat effect on GBM6 stem like cells. (**A**) Representative images of GBM6 clones migration using the transwell migration assay (crystal violet staining, magnification 100×). GBM6 sh0 cells treated or not (control) with vorinostat at various concentration. At least three independent experiments were performed, bar = 200 µm. (**B**) Analysis of proteins level expression by Western blot of GBM6 sh0 cells treated after 18 hours of treatment with vorinostat at various concentrations. In parallel effect of shEB1. Ratios (%) EB1/GAPDH and EB3/GAPDH, from at least three independent experiments are presented under the blots.
